# Factors influencing voluntary premarital medical examination in Zhejiang province, China: a culturally-tailored health behavioral model analysis

**DOI:** 10.1186/1471-2458-14-659

**Published:** 2014-06-28

**Authors:** Yaming Gu, Lu Li, Chi Zhou, Tingzhong Yang, Hengjin Dong

**Affiliations:** 1 Institute of Social Medicine & Family Medicine, Zhejiang University, Hangzhou 310058, China; 2 School of Health management, Hangzhou Normal University, Hangzhou 310058, China; 3 Institute of Public Health, Heidelberg University, Heidelberg 69120, Germany

**Keywords:** Premarital medical examination, Health belief model, Theory of reasoned action, Multilevel analysis model, Structural equation modeling, Marlowe-Crowne social desirability scale

## Abstract

**Background:**

Premarital medical examination (PME) compliance rate has dropped drastically since it became voluntary in China in 2003. This study aimed to establish a prediction model to be a theoretic framework for analyzing factors affecting PME compliance in Zhejiang province, China.

**Methods:**

A culturally-tailored health behavioral model combining the Health Behavioral Model (HBM) and the Theory of Reasoned Action (TRA) was established to analyze the data from a cross-sectional questionnaire survey (n = 2,572) using the intercept method at the county marriage registration office in 12 counties from Zhejiang in 2010. Participants were grouped by high (n = 1,795) and low (n = 777) social desirability responding tendency (SDRT) by Marlowe-Crowne Social Desirability Scale (MCSDS). A structural equation modeling (SEM) was conducted to evaluate behavioral determinants for their influences on PME compliance in both high and low SDRT groups.

**Results:**

69.8% of the participants had high SDRT and tended to overly report benefits and underreport barriers, which may affect prediction accuracy on PME participation. In the low SDRT group, the prediction model showed the most influencing factor on PME compliance was behavioral intention, with standardized structural coefficients (SSCs) being 0.75 (P < 0.01), and the intention was positively determined by individual’s attitude toward PME (SSCs = 0.48, P < 0.01) and subjective norms (SSCs = 0.22, P < 0.01) and negatively determined by perceived threat (SSCs = -0.08, P = 0.028). Attitudes and subjective norms were more crucial predictors for PME compliance than perceived threat (SSCs = 0.36, 0.269, and -0.06, respectively). County environmental factors played a role in PME compliance while less influential than behavioral determinates (16% vs. 84% in across factor variance partition coefficient).

**Conclusions:**

PME compliance might be influenced by demographic, behavioral, and social environmental factors. The verified prediction model was tested to be an effective theoretic framework for the prediction of factors affecting voluntary PME compliance. It also should be noted that internationally available behavioral theories and models need to be culturally tailored to adapt to particular populations. This study has provided new insights for establishing a theoretical model to understand health behaviors in China.

## Background

Premarital medical examination (PME) has proved to be an effective measure to prevent diseases such as syphilis, hemoglobinopathies, human immunodeficiency virus (HIV) and hepatitis B [1,2]. Mandatory PME refers to policies that make certain medical examinations a necessary condition for marriage, especially in which diseases are endemic for various legal and cultural reasons, and other educational and cost-effectiveness factors [1-6]. For example, in the United Arab Emirates, screening for syphilis, hepatitis B, and HIV in new marriages is required by law [2], while in Nigeria, religious factors drive HIV screening [6]. However, voluntary PME is more dominant form than the mandatory form [2,7-9] worldwide probably due to ethical considerations and its overtones of eugenics. In China, PME used to be compulsory by the marriage law [10]. In 2003, China introduced new regulations on marriage registration and PME became voluntary. Despite the policy of free of charge for PME service in some provinces in China, the number of couples undergoing PME has dropped drastically [11], from 94.3% in 2003 to 1.6% in 2004 and 40% in 2009 in Zhejiang province. Many efforts [11-13] to explain the determinants for PME compliance have attested that they are multifaceted and complex. Therefore, a proper theoretical framework is needed. Health behavioral theories and models are attempts to explain the reasons behind health behavioral patterns. These theories cite environmental, personal, and behavioral characteristics as the major factors in behavioral determination [14,15].

Health Behavioral Model (HBM) is one of the most widely used models for studying individuals’ participation in medical screening [15-17]. It was developed in the 1950s to explain "the widespread failure of people to accept disease preventives or screening tests for the early detection of asymptomatic disease" [18,19]. Later, it was extended to patients’ responses to symptoms and adherence to prescribed medical regimens [20,21]. This model postulates that, for individuals to participate in screening, they must have a belief about [15,22] the possibility of getting a disease (perceived susceptibility), the seriousness of contracting an illness and its consequences (perceived severity), the combination of susceptibility and severity labeled as perceived threat, the efficacy of taking the advised action to reduce the threat of illness (perceived benefits), and reducing the tangible and psychological impediments to undertaking the recommended behavior (perceived barriers) [15,22].

Another frequently adopted health behavioral theory is the Theory of Reasoned Action (TRA), which was developed in 1975 by Fishbein and Ajzen to understand relationship between attitudes, intentions, and behaviors [15]. According to TRA, the most proximate determinant of behavior is behavioral intention, which is determined by attitude toward performing the behavior and by subjective norm. Attitude, defined as "overall evaluation of the behavior", is determined by individual’s belief that behavioral performance is associated with certain outcomes (behavior beliefs) and evaluations of behavioral outcomes. The subjective norm is determined by individual’s belief about each important referent approval or disapproval of the behavior (normative belief) and individual’s motivation to comply with each referent (motivation to comply).

**Figure 1:**
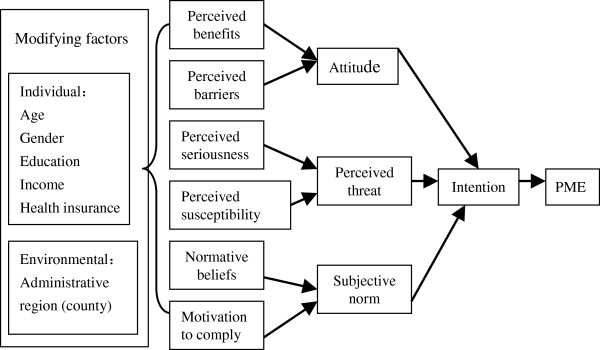
Prediction model of PME compliance.

Although health behavioral theories and models have been widely used, there have been concerns and problems that limit their effectiveness, especially in traditional, non-Western populations [23]. First, variables of investigation are substantially overlapped between different theories [24]. For example, the constructs of perceived benefits and barriers from HBM are close to the ones of attitudes from TRA. Cues to action from HBM are similar to subjective norm from TRA [14], whereas perceived threat and self-efficacy of HBM and behavioral intention of TRA are exclusive [25]. Those overlaps may affect the accuracy in analysis of theoretical integration [26,27]. Second, in practice, it is unclear how these theories are selected and how different theories are integrated. Third, it is also challenging to clearly clarify the methods for measuring theoretical constructs and analyzing data [28,29]. Moreover, available behavioral models were mainly based on western society. Therefore theoretical integration is recommended [14,25] to form a culturally-tailored model. In addition, most of the published studies [1,9,30-32] on voluntary PME were descriptive rather than theoretical, especially in China. The purpose of this study was to develop a new model to be a theoretic framework for the prediction of factors affecting voluntary PME participation. In our prediction model (Figure 1), we combined the widely-used HBM and TRA and conducted a PME compliance survey in which the prediction model was tested.

## Methods

### Participants, questionnaires, and data collection

**Figure 2:**
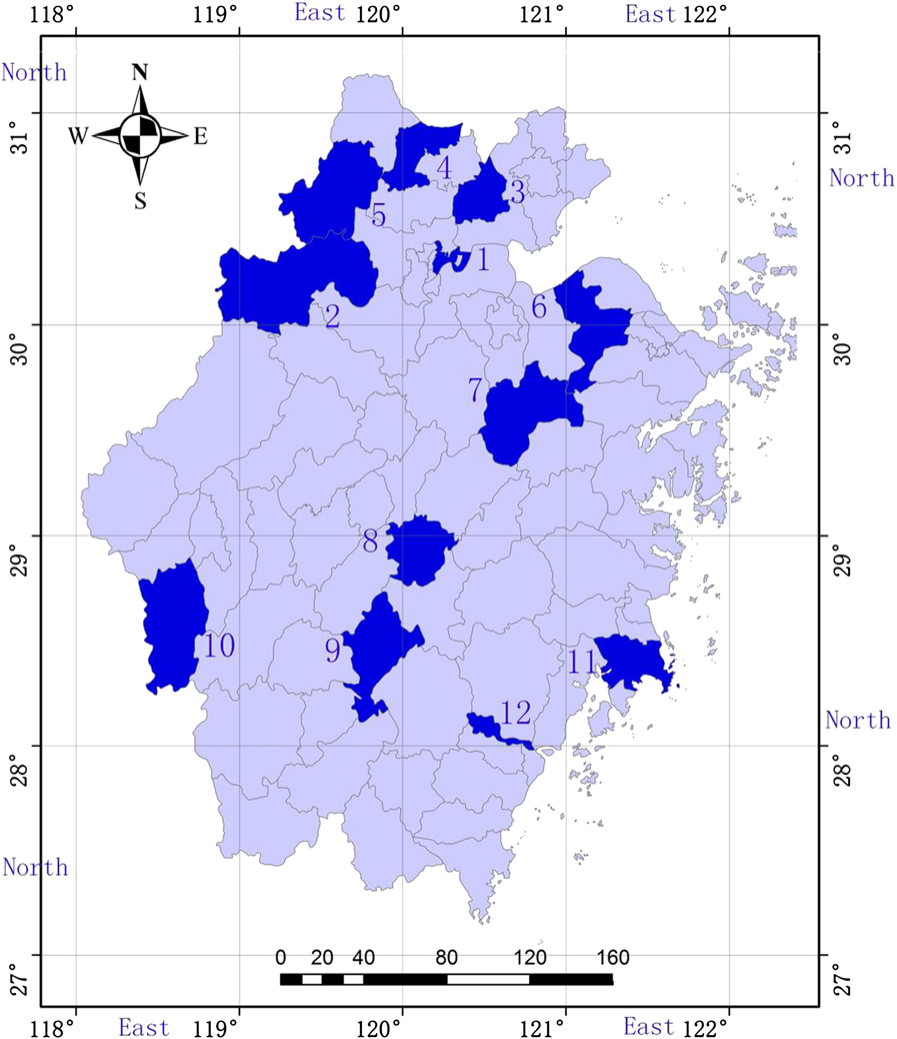
The map of Zhejiang province and 12 countries sampled.

A comprehensive survey of PME compliance was conducted during September to December 2010 in Zhejiang province, China, and the participants, questionnaires, and data collection were described in a previous study [33]. Briefly, we first chose 12 counties from 90 counties in Zhejiang province to be survey sites (Figure 2). Since all to-be married couples must go to the county marriage registration office for marriage certificate and voluntarily register for PME, so the intercept survey at the county marriage registration office was used to recruit a total of 2,572 subjects who were willing to participate in the study and had signed the consent form among of 116,494 to-be married couples in these 12 counties in 2010. Participants took about 10 minutes to complete a red 4-page high quality printed questionnaire, and received complimentary tokens like a piece of toothpaste and a towel valued at 10yuan RMB. Based on the previous work [33], a questionnaire covered socio-demographic characteristics and behavioral determinants was used and 24 trained investigators (15 nurses and 7 maternal/child health care doctors) from these 12 county’s maternal and children hospitals conducted the survey and collected the data.

The questionnaire also included social desirability responding tendency (SDRT) determinants, which was specifically designed for the current study. SDRT determinants were defined as the tendency of individuals to present themselves in a favorable response with respect to the social norm and standards [34]. We hypothesized that it is applicable to measure influences of individual behavioral determination on PME compliance. The Marlowe-Crowne Social Desirability Scale (MCSDS) is the most commonly used tool designed to assess SDRT [35], with 33 true-false items in its original version [36]. In this study, we adopted a shortened 13-item version revised by Reynolds [37], and the reliability and validity of its Chinese version were tested by Tao P [38]. The scale scores from 0 to 13, with a high score indicating a high SDRT. With a cut-off point of 7, we differentiated participants with high SDRT from ones with low SDRT. All data were collected with institutional review board approval from the Ethics Committee of Zhejiang University in conformity with all national laws and provincial regulations.

### Data analyses

Data were processed by EpiData 3.1, and we applied multivariable multilevel logistic regression [39] using MLwiN2.02 to assess the independent predictive abilities of socio-demographic, individual behavioral, and county environmental influences on PME compliance. Independent variables included socio-demographic and medical history characteristics; behavioral determinants including perceived benefits, perceived barriers, attitude, perceived seriousness, perceived susceptibility, normative beliefs, and motivation to comply; and county environmental determinants as a whole. The dependent variable was PME compliance, assigned with 1 for compliance and 0 for incompliance. The stepwise backward Wald method [40] allowed identification of the variables that are significantly associated with the outcome (p < 0.05). Adjusted odds ratios (ORs) were calculated for all variables. In the multilevel logistic regression model, all behavioral determinants were recorded from a 5-gradescale into dichotomous variables of means for corresponding determinants with a cut-off point of 2. The probability level was set at p < 0.05 to reach statistical significance.

**Figure 3:**
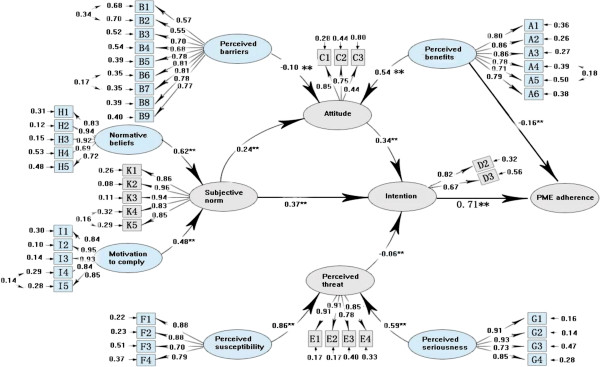
**Full model for predicting premarital medical examination (n = 2572).** Note: ** P < 0.01; Circles represented latent factors, rectangles represented observed variables, and arrows represented standardized structural coefficients. Standardized factor loadings leading from latent variables to observed variables indicate the degree to which an observed variable is influenced by a particular latent variable. The number next to observed variables is the error variance for each latent variable attributable to corresponding observed variables. Over all model fit *X*^*2*^/df = 4.12, RMSEA = 0.102, CFI = 0.90.

We used Structural Equation Modeling (SEM) to assess the adequacy of the prediction model (Figure 1). LISREL 8.71 for Windows was used to determine whether the data fit the model. We first pre-tested the model with a sample of 598 participants from 3 counties in the pilot study between April and June 2010. Based on the pretesting findings, we added two pathways to the prediction model (Figure 3), one from subjective norms to attitude and the other from perceived benefits to PME compliance for the reason of big modification indices (MI = 48.5 and 27.3 respectively, both p < 0.001) in SEM. We then applied the revised model to all participants (n = 2,572) to validate the model. At last, the finalized model was used to investigate influences on PME compliance in the high (n = 1795) and low (n = 777) SDRT groups. Criteria of goodness-of-fit statistics included ratios of *X*^2^ values to the degrees of freedom of between 2 and 5, root mean square error of approximation (RMSEA) equal or less than 0.08, and comparative fit index (CFI) equal or more than 0.9 [41].

## Results

### Socio-demographics of participants

The 2,572 participants aged from 19 to 59 years old (mean = 26.3; SD = 3.9), 50% were female, 59.2% (n = 1,523) described themselves as agriculturally registered permanent residents, and 29.3% (n = 753) had other check-ups during the last 6 months. The characteristics of education, occupation, income, health insurance, and type of current marriage registration between the participants in PME compliance group (n = 2,007) and those in PME incompliance group (n = 565) were described in Table 1. The mean score and SD of behavioral determinants were described as perceived benefits 4.46 (SD = 0.58), perceived barriers 2.51 (SD = 0.84), attitude 4.21 (SD = 0.66), perceived susceptibility 1.62 (SD = 0.61), perceived seriousness 3.97 (SD = 1.04), normative beliefs 3.68 (SD = 0.99), and motivation to comply 3.87 (SD = 0.90).

**Table 1:** Socio-demographic characteristics of the participants (n = 2572)

**Socio-demographic factors**	**Compliance n (%)**	**Incompliance n (%)**
Education		
Primary school and below	10 (62.5)	6 (37.5)
Secondary school	148 (82.2)	32 (17.8)
Senior high school	198 (85.0)	35 (15.0)
Junior college	161 (81.3)	37 (18.7)
Undergraduate college	93 (68.9)	42 (31.1)
Master degree and above	7 (46.7)	8 (53.3)
Occupation		
Government departments and institutions	232 (64.4)	128 (35.6)
Enterprises	641 (75.1)	212 (24.9)
Businessman	396 (84.4)	73 (15.6)
Agricultural farmer	74 (91.4)	7 (8.6)
Non-agricultural farmer	45 (76.3)	14 (23.7)
Migrant workers	207 (82.1)	45 (17.9)
Student	3 (33.3)	6 (66.7)
Urban unemployed	19 (73.1)	7 (26.9)
Other	390 (84.2)	73 (15.8)
Incoming per month		
Less than 400 RMB	80 (83.3)	16 (16.7)
401-1000 RMB	79 (76.7)	24 (23.3)
1001-2000 RMB	730 (80.6)	176 (19.4)
2001-3000 RMB	608 (80.2)	150 (19.8)
3001-5000 RMB	349 (71.5)	139 (28.5)
5001-10000 RMB	113 (72.9)	42 (27.1)
More than 10000	48 (72.7)	18 (27.3)
Medical insurance		
Urban employees	656 (68.8)	297 (31.2)
Urban residents	124 (73.8)	44 (26.2)
New rural cooperative one	814 (88)	111 (12)
Commercial one	59 (74.7)	20 (25.3)
Other	89 (78.1)	25 (21.9)
None	265 (79.6)	68 (20.4)
Type of current marriage registration		
Firstly married (unpregnant)	1464 (77.7)	419 (22.3)
Firstly married (pregnant)	472 (84.0)	90 (16.0)
Remarried	71 (55.9)	56 (44.1)

### Socio-demographic influences on PME compliance

Socio-demographic influences on PME compliance were processed by the multivariable multilevel logistic regression (Table 2). Factors that were not significantly related to PME compliance included age, household registration, education, monthly income per capita, and medical insurance, whereas occupation was significantly related to PME compliance as farmers and businessmen were more compliant in taking PME than governmental and institutional workers (OR = 3.02, 95% CI: 1.44 ~ 6.34; OR = 2.02, 95% CI: 1.42 ~ 2.88, respectively). Compared with the first time-married unpregnant participants, the first time-married pregnant ones were more likely to comply with PME (OR = 1.51, 95% CI: 1.14-2.00), while the remarried ones were less likely (OR = 0.35, 95% CI: 0.23-0.51).

**Table 2:** Multivariable multilevel logistic regression of PME compliance among the participants (n = 2572)

	**Estimate ± standard error**	**Odds ratio (95% *CI*)**	***P *** **value**	^**b**^**VPC (%)**
Individual level fixed effects	—	—	—	—
Intercept	-0.669 ± 0.465	0.51 (0.21,1.27)	0.151	16.89
Occupation (Government departments and institutions^a^)	—	—	—	—
Enterprises	0.484 ± 0.151	1.62 (1.21,2.18)	0.001	17.45
Businessman	0.704 ± 0.180	2.02 (1.42,2.88)	<0.001	17.53
Agricultural farmer	1.106 ± 0.378	3.02 (1.44,6.34)	0.003	16.75
Non-agricultural farmer	0.582 ± 0.366	1.79 (0.87,3.67)	0.111	17.16
Migrant workers	0.397 ± 0.212	1.49 (0.98,2.25)	0.061	16.97
Student	-0.259 ± 0.741	0.77 (0.18,3.30)	0.727	15.40
Urban unemployed	0.624 ± 0.561	1.87 (0.62,5.60)	0.266	17.43
Other	0.555 ± 0.181	1.742 (1.22,2.48)	0.002	16.83
Other check-ups during the last 6 month (None^a^)	-0.275 ± 0.114	0.76 (0.61,0.95)	0.016	16.25
Type of current marriage registration (First time-married unpregnant^a^)	—	—	—	—
Time-married pregnant	0.410 ± 0.144	1.51 (1.14,2.00)	0.004	17.46
Remarried	-1.063 ± 0.198	0.35 (0.23,0.51)	<0.001	13.45
Perceived benefits (disagree^a^)	0.841 ± 0.345	2.32 (1.18,4.60)	0.015	17.39
Perceived barriers (disagree^a^)	-0.337 ± 0.118	0.71 (0.57,0.90)	0.004	15.93
Attitude (disagree^a^)	0.523 ± 0.217	1.69 (1.10,2.58)	0.016	17.58
Normative beliefs (disagree^a^)	0.541 ± 0.125	1.72 (1.34,2.20)	<0.001	17.45
Random effects variance	—	—	—	—
County level	0.994 ± 0.419	—	0.018	—
Individual level	1	—	—	—

^a^Reference group; ^b^VPC: Variance partition coefficient.

### Environmental influences on PME

Using multivariable multilevel logistic regression allowed us to simultaneously examine both the effects of factors at the participant’s individual level, such as socio-demographic factors and behavioral determinants, and the effects of factors at the county environment on PME compliance. Taking the county environment as an undivided factor, we applied the random effects model [40] of multivariable multilevel logistic regression to examine whether county environmental influence as a whole was significant to PME compliance and how much it could affect (Table 2). The difference on PME compliance was significant across counties (*P* = 0.018), indicating that county environment as a whole did affect PME compliance. VPC (variance partition coefficient) across each factor, such as occupation, perceived benefits, perceived barriers, and attitude, was between 15.40% and 17.58%, indicating that about 16% of variation in PME compliance was attributed to county environmental influence as a whole, and about 84% of variation in PME compliance was attributed to individual level factors, i.e., socio-demographic factors and behavioral determinants.

### Behavioral determinants’ influences on PME compliance

Direct and indirect effects of behavioral determinants on PME compliance in the prediction model were shown in Figure 1 and the confirmatory results of the standardized structural coefficients (SSCs) in all pathways for all participants (n = 2,572) by SEM were illustrated in Figure 3. The behavioral intention was the most proximate determinant of PME compliance (SSCs = 0.71, P < 0.01), which was determined by individuals’ attitude toward PME (SSCs = 0.34, P < 0.01), their subjective norm (SSCs = 0.37, P < 0.01), and perceived threat (SSCs = -0.06, P < 0.01). Subjective norm directly affected intention to PME compliance (SSCs = 0.37 × 0.71 = 0.26, P < 0.01) and also indirectly enhanced intention by influencing individuals’ attitude toward PME (SSCs = 0.24 × 0.34 × 0.71 = 0.06), with the total correlation from subjective norm to PME compliance was 0.32 (SSCs = 0.26 + 0.06 = 0.32). It needs to be noted that, compared with the criteria of goodness-of-fit statistics, the confirmatory results were good in general for the indices of the prediction model (*χ*2/df = 4.12, RMSEA = 0.102, CFI = 0.90) and 53.0% of variance in PME compliance was explained by the model in statistic. The SSCs of the two pathways added to the prediction model were r = 0.24 (P < 0.01) for the pathway from subjective norm to attitude and -0.16 (P < 0.01) for the one from perceived benefits to PME compliance, showing a possible existence of the 2 pathways in logic.

### Social desirability responding tendency (SDRT)’s influences

**Figure 4:**
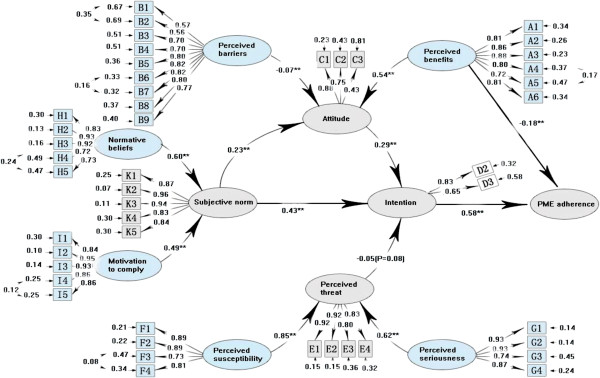
**Full model for predicting premarital medical examination among high social desirability responding tendency group (n = 1795).** Note: ** P < 0.01; Circles represented latent factors, rectangles represented observed variables, and arrows represented standardized structural coefficients; Standardized factor loadings leading from latent variables to observed variables indicate the degree to which an observed variable is influenced by a particular latent variable. The number next to observed variables is the error variance for each latent variable attributable to corresponding observed variables. Over all model fit: *X*^2^/df = 4.21, RMSEA = 0.108, CFI = 0.88.

**Figure 5:**
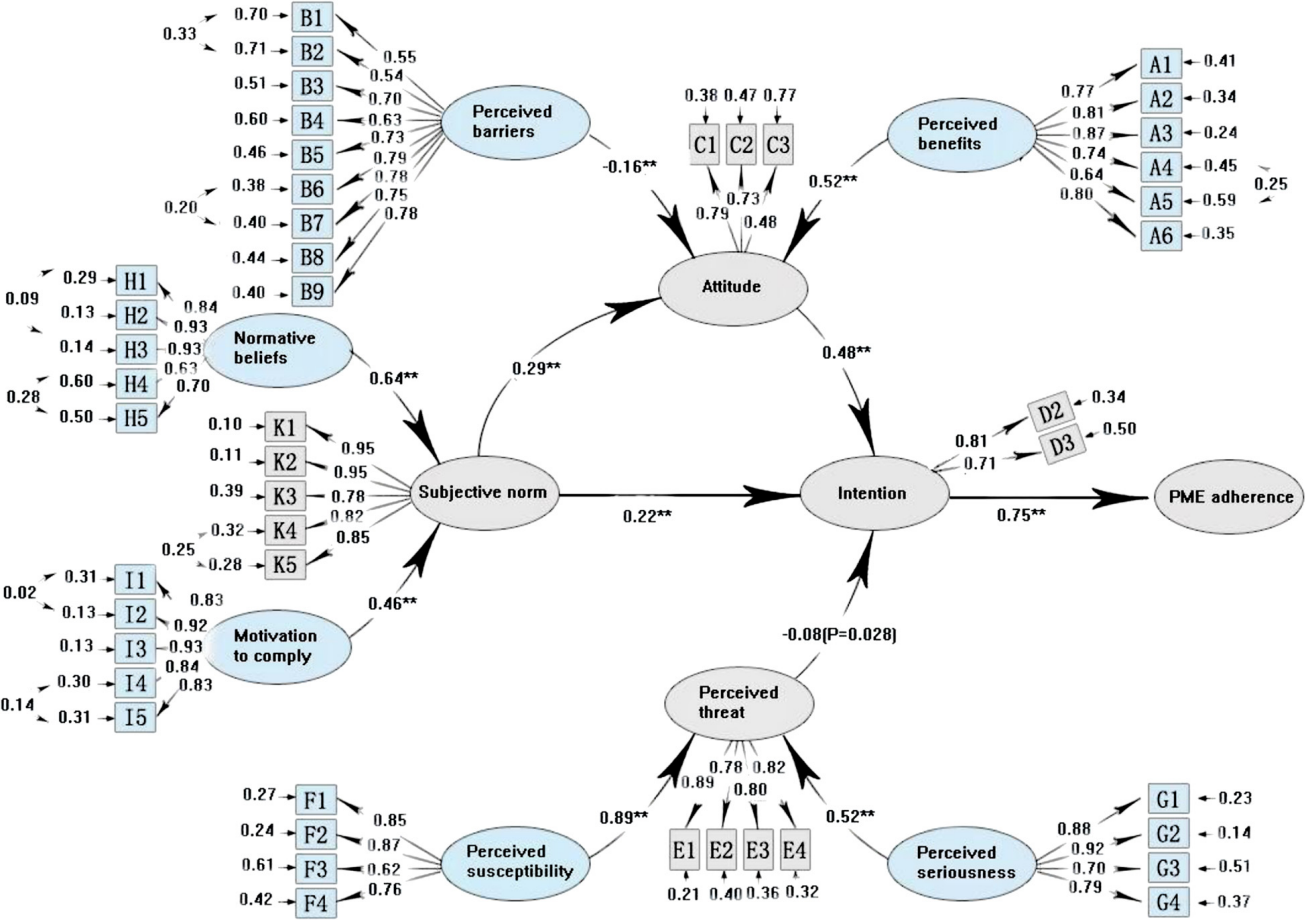
**Full model for predicting premarital medical examination among low social desirability responding tendency group (n = 777).** Note: ** P < 0.01; Circles represented latent factors, rectangles represented observed variables, and arrows represented standardized structural coefficients; Standardized factor loadings leading from latent variables to observed variables indicate the degree to which an observed variable is influenced by a particular latent variable. The number next to observed variables is the error variance for each latent variable attributable to corresponding observed variables. Over all model fit: *X*^2^/df = 4.03, RMSEA = 0.098, CFI = 0.91.

A total of 1,795 (69.8%) participants had high SDRT while 777 (30.2%) had low SDRT. In order to investigate the SDRT influence on PME compliance, we applied the revised model (Figure 3) to both high (Figure 4) and low (Figure 5) SDRT groups. Compared with the criteria of goodness-of-fit statistics, the confirmatory results of both groups were good in general for the indices of the prediction model (*χ*2/df = 4.21, RMSEA = 0.108, CFI = 0.88 for high SDRT; *χ*2/df = 4.03, RMSEA = 0.098, CFI = 0.91 for low SDRT). Differences were observed between the high and low SDRT groups. First, the prediction model displayed a higher power to explain the variance of PME compliance in the low SDRT group (56%) than in the high SDRT group (39%). Second, in the low SDRT group, the standardized structural coefficients were statistically significant (r = -0.08, P = 0.028) for the pathway from perceived threat to intention and not statistically significant (Z = -0.57, P = 0.28) for the pathway from perceived benefits to PME compliance, which is in accordance with the prediction model (Figure 1). However, in the high SDRT group, the SSCs were not statistically significant (Z = -1.89, p = 0.08) for the pathway from perceived threat to intention and statistically significant (SSCs = -0.18, p < 0.01) for the pathway from perceived benefits to PME compliance, which is in contradiction with the prediction model (Figure 1). Therefore, we concluded that our prediction model clearly explained the SEM of PME compliance in the low SDRT group. The results indicated that the illusive direct pathway from perceived benefits to PME compliance (MI = 27.3, p < 0.001; all participants, n = 2,572; Figure 3) was caused by SDRT, the tendency of participants to present themselves in a favorable response with respect to the social norm and standards of PME.

### The verification of the prediction model

Our prediction model for the investigation of factors that affect voluntary PME compliance was well verified in the low SDRT group (Figure 5). First, the most influencing factor affecting the actual participation in PME was behavioral intention, with SSCs of 0.75 (P < 0.01), which was positively determined by individuals’ attitude toward PME (SSCs = 0.48, P < 0.01) and subjective norm (SSCs = 0.22, P < 0.01), and negatively determined by perceived threat (SSCs = -0.08, P = 0.028). Second, PME compliance was directly affected by subjective norms (SSCs = 0.22 × 0.75 = 0.165, P < 0.01) and indirectly enhanced by influencing individuals’ attitude toward PME (SSCs = 0.29 × 0.48 × 0.75 = 0.104), with the total correlation from subjective norm to PME compliance being 0.269 (r = 0.165 + 0.104 = 0.269). Third, the awareness of the benefits of PME and decreased perception of barriers could enhance the positive attitudes toward PME and eventually change PME compliance. Both of normative belief and motivation to comply could indirectly enhance PME compliance through the media of subjective norm and intention. Lastly, perception of the susceptibility and the severity had a weak indirect negative effect on PME compliance through the function of perceived threat (SSCs: -0.05 and -0.03, respectively).

## Discussion

### The prediction model

In the present study, we established a prediction model that combined the widely-used HBM and TRA behavioral models as a theoretic framework for the investigation of factors affecting voluntary PME participation in Zhejiang province, China. In this combined model, we excluded and retained some constructs from HBM and TRA according to their relevance to traditional non-Western populations like China [23].

First, we excluded the construct of self-efficacy from HBM. Based on frequency, health behaviors can be classified into ongoing or repeated behaviors (i.e., seat belt use), intermittent behaviors (i.e., annual influenza vaccination), and circumscribed preventative action (i.e., a new vaccine or a new screening test) [28]. The construct of self-efficacy is more useful in ongoing behaviors and intermittent behaviors than in circumscribed preventative ones [15,28]. Health behaviors of interest in this study are of circumscribed preventative action, so the construct of self-efficacy was not felt to add explanatory power and thus not included. Actually, Champion and Skinner suggested that self-efficacy was never explicitly incorporated into early version of HBM because of its focus on circumscribed preventive actions [42].

Second, the construct of subjective norm in TRA, instead of cues to action in HBM, was retained in the combined model. Although some formulation of the HBM included the construct of cues to action, it is diverse in nature and has been problematic to identify and measure [26], especially in explanatory studies [28]. Furthermore, Chinese culture emphasizes the norms of reciprocity [43] and attending to others [44], which contribute to interpersonal behavior patterns and rules of exchange. They are heavily shaped by hierarchically structured network of social relations, the public nature of obligations, and self-conscious manipulation of face [42]. Thus, Chinese social behaviors are more easily influenced by opinions and behaviors from others, especially by those from powerful figures and important referents, compared with ‘independent self’ societies [44].

Third, the construct of intention was retained in the combined model because it is an integral part of TRA and doesn’t overlap the constructs of HBM. Similar attempts have been made in many other studies [26,27,45-47]. Fourth, socio-demographic factors were also taken into account because they have been shown to influence medical screening behaviors such as mammography and genetic testing for cancer risk [48-50]. Furthermore, socio-demographic variables were included into analysis because they were thought to have an indirect effect on health behaviors by influencing the theoretical constructs [42].

### The verified model and factors affecting voluntary PME participation

Our PME compliance prediction model included 10 behavioral determinants (Figure 1) and its relevance and accuracy were verified by SEM in the low SDRT group (Figure 5). The results support the verified model as a theoretic framework for the prediction of factors affecting voluntary PME participation. Couples who believed that PME was effective and had no tangible and psychological impediments (attitudes), who believed that important referents advocated PME and encouraged couples themselves to comply with it (subjective norms), and who felt susceptible and were serious about related disease (perceived threat) were more likely to receive PME than others otherwise. Of these three beliefs (attitudes, subjective norms, and perceived threat), attitudes and subjective norms were more crucial predictors to PME participation than perceived threat (SSCs = 0.36, 0.269, and -0.06, respectively). It is consistent with previous health-related behaviors studies [51-53]. Moreover, attitude was more important than subjective norm to PME compliance. Trafimow and Finlay measured attitudes and subjective norms for 30 behaviors and showed that 29 were more under attitudinal than normative control [53,54]. Interestingly, subjective norms were found to be more influential in this study than in some western health-related behaviors studies [53,54]. It may be explained by the fact that Chinese social behaviors are greatly influenced by others [44]. In particular, we suggest that physicians and leaders of Village Women Society may play an important role in promoting PME compliance in China.

In the current study, behavioral intention was found to be a good predictor of PME compliance according to SEM results. Moreover, SSCs of behavioral intention to PME compliance were higher in the low SDRT group than those in the high SDRT group (SSCs = 0.75 vs. 0.58), indicating that the prediction power became greater when socially desirable response bias was controlled. Other researchers also reported that behavioral intention provided a moderate to strong prediction of behaviors of health checkups or tuberculosis detection [45,46]. Some even suggested that it should be considered as a mediating variable between the HBM dimensions and behaviors [45,55,56]. Indeed, we found that perceived barriers, perceived benefits, perceived susceptibility, and perceived seriousness were mediated through intention. Behavioral intention can be measured repeatedly before action, which is valuable for designing and evaluating intervention procedures to foster PME compliance.

### Environmental influences on PME

In the current study, multivariable multilevel logistic regression results show that county environmental factors played a role in PME compliance, while less influential than behavioral determinates, which was confirmed by the prediction model. In China, the county environmental factors may include regional cultural features such as policies and other promotion measures for PME, which may bring participants rewards (free of charge) and convenience (one line service). Similar findings were also reported in cancer screening in other cultural environments. Fukuda found that the proportion of region-related (prefectures) variance for stomach, colon, uterine and breast cancers screening ranged from 19.5%-27.6% after considering individual variables in Japan [57]. Lian reported that nearly 15% of the colorectal cancer screening was from geographic variation in Missouri, USA [58]. Even though environmental context may influence health behaviors, they are not an explicit part of HBM and TRA when they are widely used in medical screening [15-17], which certainly will affect the prediction accuracy. Our experience indicates that culturally-tailored theoretical integration and modification of existing behavioral theories and models is necessary and practical.

### SDRT’s influences

The self-reporting questionnaire survey including psychological instruments is often susceptible to socially desirable response bias [34]. In this study, 69.8% of the participants had high SDRT and the profiles of predicting factors to PME compliance were different between the high and low SDRT groups (Figures 4 and 5), especially in the pathway from perceived threat to intention and the pathway from perceived benefits to PME compliance. This indicates that those with higher SDRT tended to overly report benefits and underreport barriers rather than giving honest responses, which potentially affects prediction accuracy in PME participation. Other studies on health-related behaviors in Chinese population reported similar findings [59,60]. It was postulated that some might feel embarrassed for not reporting the willingness to take PME [61]. We’d like to add that it is likely due to collectivism-orientation of Chinese culture. Some research also concluded that social desirability scores may be higher in collectivistic societies [62]. Thus, researches involving self-reporting need to consider biases from social desirability responses, especially in collectivistic cultural populations. In particular, a social desirability scale should be added in controlling potential biases to self-reporting questionnaire studies.

## Conclusion

Our prediction model for the investigation of factors affecting voluntary PME compliance was well verified in the low SDRT group. This model provided a solid analysis of local PME compliance behavior. We conclude that PME compliance in Zhejiang province might be influenced by demographic characteristics, behavioral determinants, and social environments. Moreover, behavioral determinants are the main factors, and the environmental factors, in particular the policies and service conveniences promoted by local governments toward PME, might greatly raise PME compliance. It is recommended that tailored health education and promotion programs to promote PME compliance in Zhejiang province should be developed based on couples’ attitudes and subjective norms on the individual level, and the environmental factors at the county level. It also should be noted that the internationally available behavioral theories and models need to be tailored to adapt to a cultural environment in which health behaviors are assessed.

This research has limitations. The county environmental influence on PME in SEM was not explored simultaneously, due to difficulties in applying multilevel structural equation model without proper computing software, and a sampling bias might be there caused by distinct provincial features of economy and culture. We believe that this study has provided new insights for establishing a theoretical model to understand health behaviors in China.

## Competing interests

The authors declare that they have no competing interests.

## Authors’ contributions

LL and GYM designed the study and completed the first draft of this article, ZC participated in formulation of data collection and analysis, and TZY and HJD revised the manuscript and made valuable suggestions on scholarly writing. All authors have read and approved the final manuscript.

## Pre-publication history

The pre-publication history for this paper can be accessed here:

http://www.biomedcentral.com/1471-2458/14/659/prepub
